# Low‐carbohydrate high‐fat weight reduction diet induces changes in human gut microbiota

**DOI:** 10.1002/mbo3.1194

**Published:** 2021-05-15

**Authors:** Madis Jaagura, Ene Viiard, Kätrin Karu‐Lavits, Kaarel Adamberg

**Affiliations:** ^1^ Center of Food and Fermentation Technologies Tallinn 12618 Estonia; ^2^ Department of Chemistry and Biotechnology Tallinn University of Technology Tallinn 12618 Estonia

**Keywords:** 16S rRNA gene, bifidobacteria, BMI, *Collinsella*, microbiome, obesity, weight loss

## Abstract

Obesity has become a major public health problem in recent decades. More effective interventions may result from a better understanding of microbiota alterations caused by weight loss and diet. Our objectives were (a) to calculate the fiber composition of a specially designed low‐calorie weight loss diet (WLD), and (b) to evaluate changes in the composition of gut microbiota and improvements in health characteristics during WLD. A total of 19 overweight/obese participants were assigned to 20%–40% reduced calories low‐carbohydrate high‐fat diet for four weeks. Protein and fat content in the composed diet was 1.5 times higher compared to that in the average diet of the normal weight reference group, while carbohydrate content was 2 times lower. Food consumption data were obtained from the assigned meals. Microbial composition was analyzed before and after WLD intervention from two sequential samples by 16S rRNA gene sequencing. During WLD, body mass index (BMI) was reduced on average 2.5 ± 0.6 kg/m^2^ and stool frequency was normalized. The assigned diet induced significant changes in fecal microbiota. The abundance of bile‐resistant bacteria (*Alistipes*, *Odoribacter splanchnicus*), *Ruminococcus bicirculans*, *Butyricimonas*, and *Enterobacteriaceae* increased. Importantly, abundance of bacteria often associated with inflammation such as *Collinsella* and *Dorea* decreased in parallel with a decrease in BMI. Also, we observed a reduction in bifidobacteria, which can be attributed to the relatively low consumption of grains. In conclusion, weight loss results in significant alteration of the microbial community structure.

## Graphical abstract

Our weight loss diet stimulated the growth of bile‐resistant fecal bacteria and reduced the levels of bacteria associated with inflammation. The appointed dietary regime introduced significant changes in the fecal microbiota of the test group compared to reference group levels. A high intake of vegetables and a low intake of cereals lead to high cellulose and pectin content in the diet and normalized the stool form.

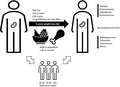

## 1 INTRODUCTION

A rise in obesity has become a major public health problem over the past three decades (Mitchell et al., 2011) due to associated chronic diseases such as type 2 diabetes (T2D), hypertension, and dyslipidemia, among others (Bays et al., 2013; Landsberg et al., 2013; Ng et al., 2014). Diet has been recognized as one of the major contributing factors in maintaining weight homeostasis (Carels et al., 2008; Keränen et al., 2009), and various dietary approaches have been described to both treat obesity and manage weight loss (Botchlett & Wu, 2018; Sacks et al., 2009).

Popular eating trends have also changed during this time with a rise in popularity of low‐carbohydrate high‐fat (LCHF), paleo, vegan, and vegetarian diets. LCHF is also very popular for the treatment of obesity due to its effectiveness when losing weight (Hall et al., 2015; Mansoor et al., 2016). To better understand the relationship between diet and human health and their associations with obesity, scientists have extensively studied the gut microbiome in recent years. Despite much work, it remains unclear how our diet and gut microbiome influence weight management and its association with obesity status.

Obesity has been linked with altered gut microbiota by additional energy harvest (Turnbaugh et al., 2006). Diet is one of the main factors that modulate gut microbiota because changes in bacterial abundance can occur very rapidly after the digested food has reached the colon (David et al., 2014). Although plant‐based diets have a significant effect on gut microbiota, animal‐based diets have been shown to have a greater impact (David et al., 2014). Nutritional profiles of the dietary patterns differ greatly in total energy and the content of macronutrients and dietary fiber (DF). Bacterial fermentation of DF has a major influence on intestinal function—the production of organic acids influences glucose and lipid metabolism, the immune system, and hormone secretion (Chambers et al., 2018). While caloric and macronutrient intake has been implicated in microbiome modulation, the effect of fiber content in weight reduction diets on the gut microbiota has been less extensively studied (Table A1). Weight reduction studies have demonstrated disparate results with regard to changes in the composition of the microbiota, which may be partially due to differences in the amount and choice of fiber in the diet. Thus, evaluating DF‐associated effects during weight loss is crucial because reducing the daily caloric intake can also lead to a reduction in DF intake (Brinkworth et al., 2009).

Generally, interventional studies that investigate the relationship between food intake and the gut microbiome are often performed by supplementing extracted and purified fiber within a regular diet, however, baseline consumption of foods can remain different (Baxter et al., 2019; Dewulf et al., 2013). To circumvent this problem, uniform diets can be designed using prepared foods for all participants, and however, it can be costly to manage food delivery and the variety of foods that can be prepared in this way is too restrictive for the subjects. Alternatively, more subject‐friendly diets can be formulated based on whole food components. To use this approach, a database of whole foods with detailed nutrient information is required, which would enable one to fine‐tune diets with specified amounts of selected nutrients. With regard to microbiota, fiber composition should be determined in detail because fibers (type and amount) drive the modulation of gut microbiota (Dewulf et al., 2013; Walker et al., 2011).

We aimed to characterize the specific fiber content and sources in both weight loss and habitual diets, to analyze the fecal microbial composition of obese and normal weight subjects, and to investigate the effect of WLD on fecal microbial consortia.

## 2 MATERIALS AND METHODS

### 2.1 Study design

A group of overweight/obese participants began a voluntary low‐calorie low‐carbohydrate high‐fat WLD interventional program to follow the appointed regime for at least four weeks. Inclusion criteria for participants included BMI of >28 kg/m^2^ in the intervention arm (maximum 25% in BMI range 28–30 kg/m^2^), BMI of >18 kg/m^2^ in the reference group, no previous history of gastrointestinal (GI) disease, no reported antibiotic use in preceding 3 months, or taking any medication known to alter bowel motility, no history of food allergies and not taking medications, not pregnant nor breast‐feeding, no specific dietary choices, and with ability to adhere to an omnivorous diet. Participant recruitment for the intervention arm was carried out in April and May 2017, and the 4‐week WLD was carried out in June 2017. Reference group recruitment was carried out in March–April 2017, and sampling in April–August 2017, described in detail previously (Adamberg et al., 2020).

### 2.2 Diet design for WLD

Assigned diet plans included 3 main meals (30 ± 5% E each) and 1 dessert (10% E) per day. Subject‐specific daily energy intake was calculated based on daily expenditure, which accounted for body weight, activity, and energy restriction of 30 ± 10%. Meals in the diet were randomly selected from specifically designed recipes, which consisted mostly of animal‐based foods (meat and dairy products) as a protein source, supplemented with vegetables, fruits, and a limited amount of cereals. Participants were provided with individual daily meal plans based on their energy needs. Subjects were asked to confirm that they had eaten the prescribed meals and they received personal assistance via phone calls if they were having any difficulties adhering to the diet. Diet plan meals were prepared by the subjects, and no additional food intake was allowed. None of the subjects consumed pre‐ or probiotics as supplements.

### 2.3 Diet analysis in the WLD and reference groups

The subjects in the reference group were instructed to record their food consumption for at least one day before sampling, described in detail previously (Adamberg et al., 2020). For calculations, meals were decomposed to ingredients based on dietary instructions in the WLD group and based on consumed foods in the reference group. Assigned WLD and reference group consumption data were analyzed for energy, macronutrient, and total DF content based on the NIHD (The National Institute for Health Development) food composition database Nutridata v6/7.

### 2.4 Diet quantification

Nutritional data were analyzed and normalized to 1,000 kcal caloric intake and presented as the mean of a group ± *SD*. Analysis of the food and diet records was carried out as previously described (Adamberg et al., 2020). Shortly, all food components were decomposed into 35 primary and 72 secondary food groups, of which 46 dietary fiber‐containing foods groups were characterized based on the fiber patterns in raw food materials to cover similar products within the same category. Meat and milk‐derived products were considered as negligible sources of DF if not containing cereals, fruit, or vegetables. Specifically, the content of arabinoxylan, β‐glucan, cellulose, inulin, lignin, and pectin was calculated for each category based on the literature data on raw foods (Bengtsson et al., 1990; Dodevska et al., 2013, 2015; Herranz et al., 1981; Holtekjølen et al., 2006; Kalala et al., 2018; Karppinen et al., 2000; nut.s, 2020). Analysis of food consumption data was carried out using custom R scripts.

### 2.5 Fecal sampling and anthropometric data collection

The subjects were asked to collect fecal samples immediately after defecation by sterile swab and to suspend the collected material in a buffer containing ammonium sulfate (40% solution), EDTA (16 mM), and sodium citrate (20 mM). With each fecal sampling, a Bristol stool scale score (BSS) was also recorded by the subjects. The samples were transported at room temperature to the laboratory and stored at −20°C before DNA extraction. For anthropometric measurements, body weight and height were measured before and after the intervention period. Height was measured to the nearest 0.5 cm and weight to the nearest 0.5 kg. BMI was calculated at the beginning and end of the study using the formula: BMI = Weight/Height^2^ [kg/m^2^]. Two fecal samples were collected sequentially before intervention and four weeks later. Participants were provided stool collection swab kits, which contained saturated ammonium sulfate solution and EDTA‐citrate buffer. Samples were delivered to the laboratory every three days and were stored at +2–6°C until extraction. Reference group samples were collected similarly, but the interval between two sampling time points varied between 41 and 121 days, on average 61 days (Adamberg et al., 2020).

### 2.6 DNA extraction and 16S rRNA gene library preparation

Fecal DNA was isolated with the use of a PureLink Microbiome DNA Isolation Kit (Thermo Fisher Scientific, US, CA, Carlsbad) according to the manufacturer's instructions. Universal primers: S‐D‐Bact‐0341‐b‐S‐17 (5′TCGTCGGCAGCGTCAGATGTGTATAAGAGACAGCCTACGGGNGGCWGCAG) and S‐D‐Bact‐0785‐a‐A‐21 (5′ GTCTCGTGGGCTCGGAGATGTGTATAAGAGACAGGACTACHVGGGTATCTAATCC) were used for PCR amplification of the 16S rRNA gene V3‐4 region (Klindworth et al., 2013). The mixture of amplicons was sequenced on Illumina MiSeq with the use of v2 reagents yielding 2 × 250 bp paired‐end reads (Estonian Genome Centre, Estonia). On average, 84,324 reads per sample were obtained for the intervention group and 82,639 reads per sample for the reference group.

### 2.7 Taxonomic profiling of the sequencing data

DNA sequence data were analyzed using BION‐meta according to the author's instructions (https://box.com/v/bion). First, sequences were cleaned at both ends using a 99.5% minimum quality threshold for at least 18 of 20 bases for 5′‐end and 28 of 30 bases for 3′‐end, then joined, followed by removal of shorter contigs than 350 bp. Sequences were cleaned from chimeras and clustered by 95% oligonucleotide similarity (k‐mer length of 8 bp, step size 2 bp). Lastly, consensus reads were aligned to the SILVA reference 16S rDNA database (v123) using a word length of 8 and a similarity cut‐off of 90%. All mapped taxa with relative abundance <0.0001 were discarded and considered as potential noise.

### 2.8 Statistical analysis

Based on sample size calculations, we estimated that with 17 participants, the study would have more than 80% power to detect a significant difference among weight loss study groups, assuming a mean BMI reduction by 3 kg/m^2^, with a mean BMI and standard deviation of 35 and 3.2 kg/m^2^, respectively, at an alpha level of 5%.

Statistical analysis included bacteria with average colonization frequency >70% and average abundance >0.001. Analysis of data was carried out in the R statistical programming language, version 3.5.0 (R Core Team, 2020). The resulting *p*‐values were corrected for multiple comparisons for each phylogenetic level using Benjamini–Hochberg correction (FDR). A corrected *p*‐value <0.1 was considered statistically significant. Unless stated otherwise, corrected *p*‐values are shown in the text.

Pairwise comparisons were evaluated using Wilcoxon signed‐rank test, for the comparison of test and reference groups Kruskal–Wallis test was applied.

To control for within‐subject variability, we used the subsequent sample pairs as within‐subject controls and compared β‐diversity before and after the intervention. This was also applied to reference group samples. The following cutoffs were used: *p < 0.05; **p < 0.01; ***p < 0.001; and ****p < 0.0001.

### 2.9 Agglomerative hierarchical clustering

Ward's agglomerative hierarchical clustering on a distance matrix was generated from a species by sample Bray‐Curtis distance matrix. The method produces a dendrogram by treating each sample as a singleton cluster, merging pairs of clusters until all clusters have been merged into one big cluster containing all samples. Ward's agglomeration method minimizes the total within‐cluster variance.

## 3 RESULTS

**FIGURE 1:**
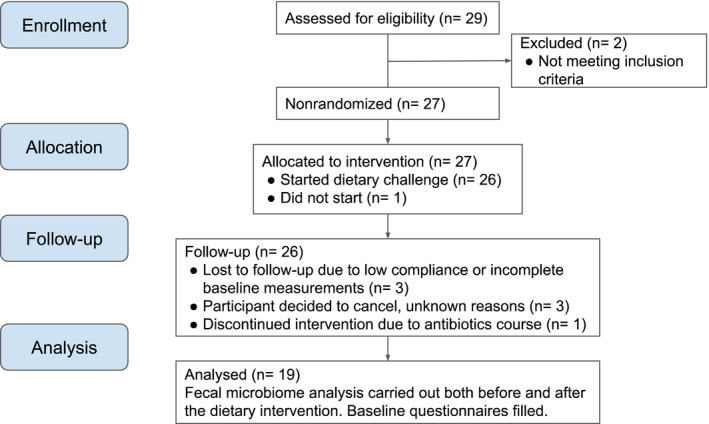
TREND flow diagram

A total of 27 overweight/obese participants were enrolled in a WLD regime. Of those, one participant did not enter the study. By the end of the study, three participants were lost due to low compliance or incomplete baseline measurements, three canceled for unknown reasons, and one discontinued the weight loss program due to an unexpected antibiotic course. Thus, a total of 19 overweight/obese participants (14 females, 5 males, aged 25 to 43) with BMI ranging between 28.9 and 44.4 kg/m^2^ successfully finished. A TREND flow diagram is displayed in Figure 1.

Additionally, 59 subjects (39 females, 20 males, aged 23–52 years) were recruited into a reference group, which was divided into three subgroups based on BMI: 18–25, 25–30, 30–39 kg/m^2^ (*N* = 33, *N* = 16, and *N* = 10, respectively) (Adamberg et al., 2020). Characteristics of both the WLD and reference groups are depicted in Table 1.

**TABLE 1:** Participant characteristics of the WLD and reference group at baseline

	WLD group		Reference group	
	Frequency	Percentage (%)	Frequency	Percentage (%)
Gender				
Female	14	73.7	39	66.1
Male	5	26.3	20	33.9
Age				
18–24			3	5.1
25–34	11	57.9	23	39.0
35–44	8	42.1	21	35.6
45–54			12	20.3
BMI				
18–25			33	55.9
25–30	1	5.2	16	27.1
30+	18	94.8	10	16.9

### 3.1 Assigned diet was high in fat, reduced BMI, and normalized bowel habits

**FIGURE 2:**
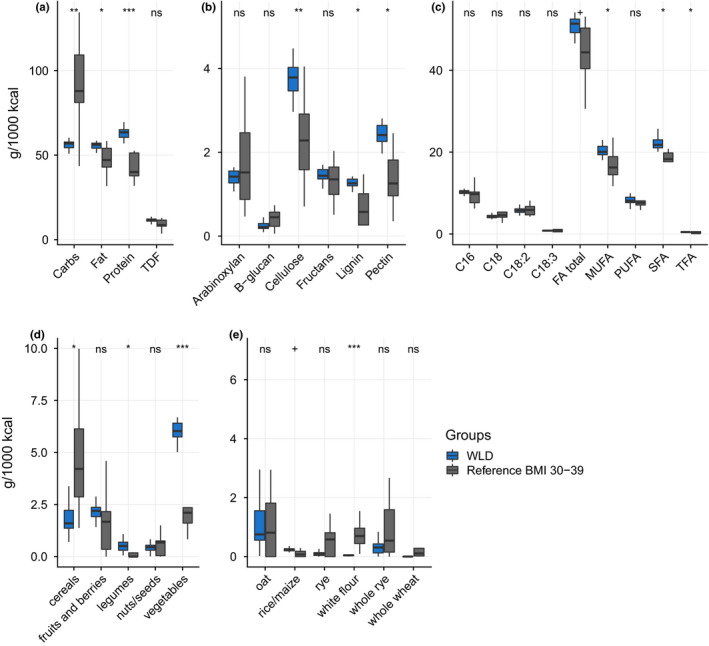
Nutritional and food intake in the WLD, and obese reference group. (a) macronutrients and total DF; (b) specific DF categories; (c) fatty acid profile; (d) main total DF sources; (e) main cereal DF sources. All values are g per 1000 kcal per day. Outliers are not shown

**FIGURE A1:**
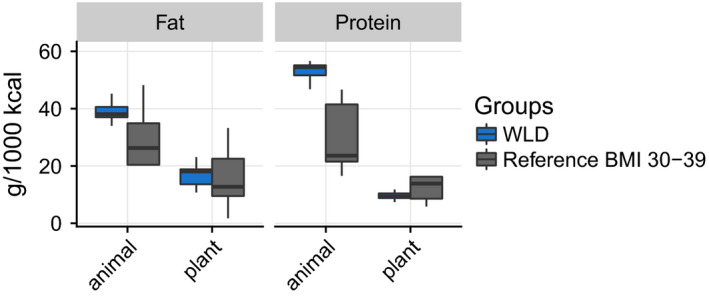
Origin of fat and protein in WLD and obese reference group

The diet used for all participants was rich in fat (55.6 ± 2.1 g / 1,000 kcal, 50% from total energy), protein (62.7 ± 3.2 g / 1,000 kcal, 25%), and low in carbohydrates (56.5 ± 3.5 g / 1,000 kcal, 23%, Figure 2). Normalized intake values per 1,000 kcal were used because the caloric intake of the participants varied greatly due to highly dissimilar body weights. Both fat and protein were mainly sourced from animal‐based foods (Figure A1). DF content was moderate (11.6 ± 1.1 g / 1,000 kcal), but it was comparable to levels in an average Estonian diet in an obese reference group (9.8 ± 4.8 g / 1,000 kcal). DF composition analysis based on food categories showed that most of the DF was cellulose and pectin (3.8 ± 0.4 and 2.4 ± 0.2 g / 1,000 kcal, respectively), which was higher than these in the obese reference group (2.5 ± 1.4 and 1.5 ± 1.0 g / 1,000 kcal, respectively). On the other hand, consumption of arabinoxylan and β‐glucan were relatively low (1.4 ± 0.2 and 0.3 ± 0.1 g / 1,000 kcal, respectively), yet a similar intake was observed in the obese reference group (1.8 ± 1.1 and 0.5 ± 0.4 g / 1,000 kcal, respectively).

DF source analysis showed that vegetables were the main source of DF (5.7 ± 0.6 g / 1,000 kcal), while intake of DF originating from cereals was very low in the WLD group (1.4 ± 0.5 g / 1,000 kcal) compared to the reference group (2.0 ± 1.2 and 3.5 ± 2.0 g / 1,000 kcal, respectively). Specifically, consumption of DF from wheat, rye, and barley during WLD was low (0.5 ± 0.4 g / 1,000 kcal). Due to limited information about food processing, the content of resistant starch (RS) was eliminated from our analysis. Low DF intake from cereals (1.4 ± 0.5 g / 1,000 kcal), starchy vegetables (0.5 ± 0.2 g / 1,000 kcal), legumes (0.6 ± 0.4 g / 1,000 kcal), and no detected increase of RS fermenting bacteria (Venkataraman et al., 2016; Ze et al., 2012) suggest that RS levels in the WLD group were low.

Analysis of fatty acids revealed that WLD was rich in saturated fatty acids (SFAs) and monounsaturated fatty acids (MUFAs) (22.0 ± 1.7 and 20.5 ± 1.7 g / 1,000 kcal, respectively) and contained similar levels of polyunsaturated fatty acids (PUFAs) (8.2 ± 1.0 g / 1,000 kcal) compared to the obese reference group (18.0 ± 4.5, 16.7 ± 3.8, and 9.0 ± 4.6 g / 1,000 kcal, respectively).

**FIGURE 3:**
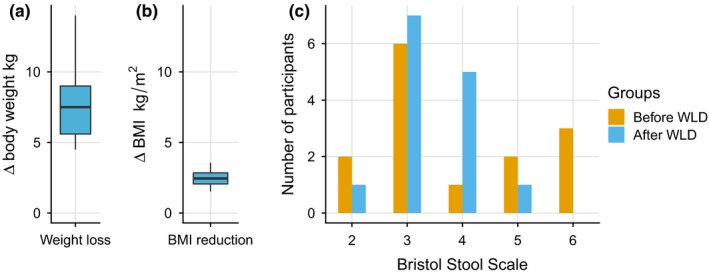
Changes in body mass (a), BMI (b), and BSS before and after the intervention (c) in the WLD group

During the intervention, the BMI and body weight were significantly reduced on average by 2.5 ± 0.6 kg/m^2^ and 7.7 ± 2.5 kg, respectively (*p* < 0.0002 for both) (Figure 3a,b).

Out of 14 participants, who filled questionnaires about gastrointestinal (GI) disturbances before and after WLD, not a single participant reported daily constipation or diarrhea and only one subject reported daily flatulence at the end of the intervention (before intervention: 1, 1, 7 subjects, respectively). A detailed description of bowel habits before and after the intervention can be found in Table A2. THE mean BSS value before WLD was 3.9 ± 1.5 and decreased to 3.4 ± 0.8 after the intervention, but in general BSS values stabilized (Figure 3c).

### 3.2 Changes in microbiota associated with food consumption and health parameters

**FIGURE A2:**
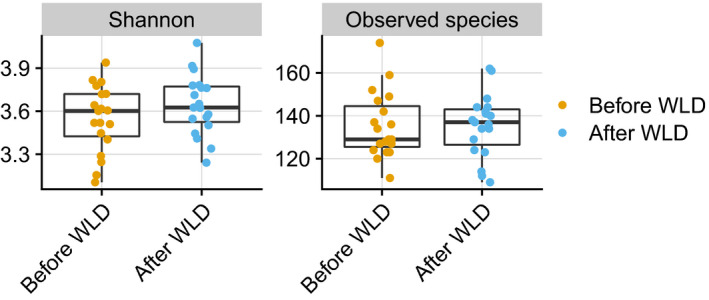
Alpha diversity and species richness

Statistically significant changes were not observed in α‐diversity (Shannon: 3.5 ± 0.2 and 3.6 ± 0.2, before and after WLD, respectively) or species richness (135.3 ± 15.3 and 135.4 ± 14.5, before and after WLD, respectively) (Figure A2).

**FIGURE 4:**
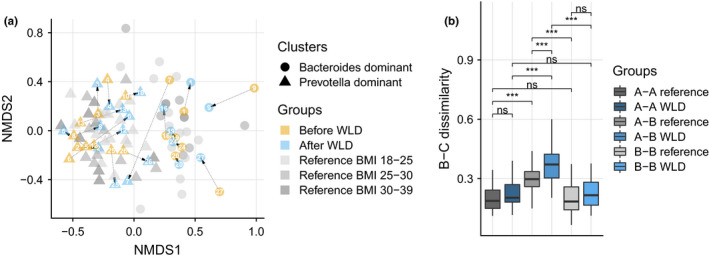
Impact of WLD on microbiota composition. A non‐metric multidimensional scaling (NMDS) ordination displaying before and after WLD samples, and reference group samples (*N* = 59). Ward's agglomerative hierarchical clustering was used to cluster all samples into two clusters, dominated by *Bacteroides* or *Prevotella* species. (a) NMDS; (b) within‐subject β‐diversity of WLD and reference samples was compared at baseline (A‐A), after intervention in the WLD group, and after a similar timeframe in the reference group (B‐B) and between two time points (A‐B). Reference—all BMI groups

**FIGURE A3:**
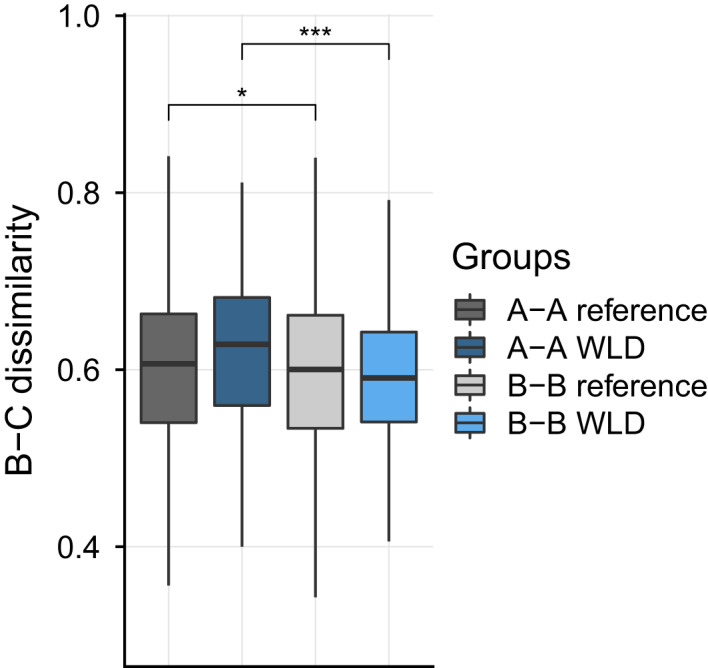
Between‐subject β‐diversity of WLD and reference samples

Bray–Curtis (B‐C) distances of species composition within subjects reveal that intervention resulted in significantly altered microbial profiles in the WLD group. Similarly, within two months, changes in the microbiota in the reference group were also significant but not as extensive as in the test group (Figure 4b). Over the study period, change in β‐diversity was observed between participants in the test and reference group (Figure A3). Overall, between‐subject dissimilarities in the test group decreased more significantly, potentially due to a similar dietary regime with comparable nutrient composition. Hierarchical clustering revealed that most of the samples before and after intervention paired together (Figure A7).

Although within‐subject B‐C distances changed significantly, both samples from the same participant before and after WLD clustered together in most cases (Figure 4a), and only one of the sample pairs switched from a *Bacteroides*‐dominated cluster to a *Prevotella*‐dominated cluster (Figure 4a).

**FIGURE 5:**
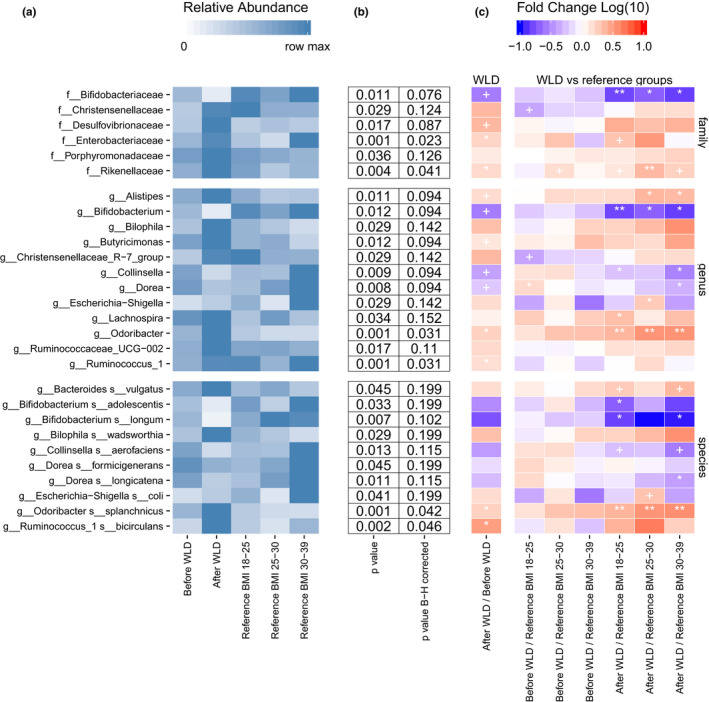
Altered taxa in response to WLD and comparison with reference groups. Statistical analysis was carried out at the family, genus, and species levels. (a) Average abundance of altered taxa. (b) uncorrected and corrected *p*‐values between before and after samples from the same subject in the WLD group. (c) Fold change after WLD vs before study and reference group abundances. The last six columns indicate the logarithmic fold change by the colors ranging from dark blue to red. B‐H corrected *p* < 0.1 (+), <0.05 (*), and <0.01 (**). See Figure A4 for full data on subject level abundances

**FIGURE A4:**
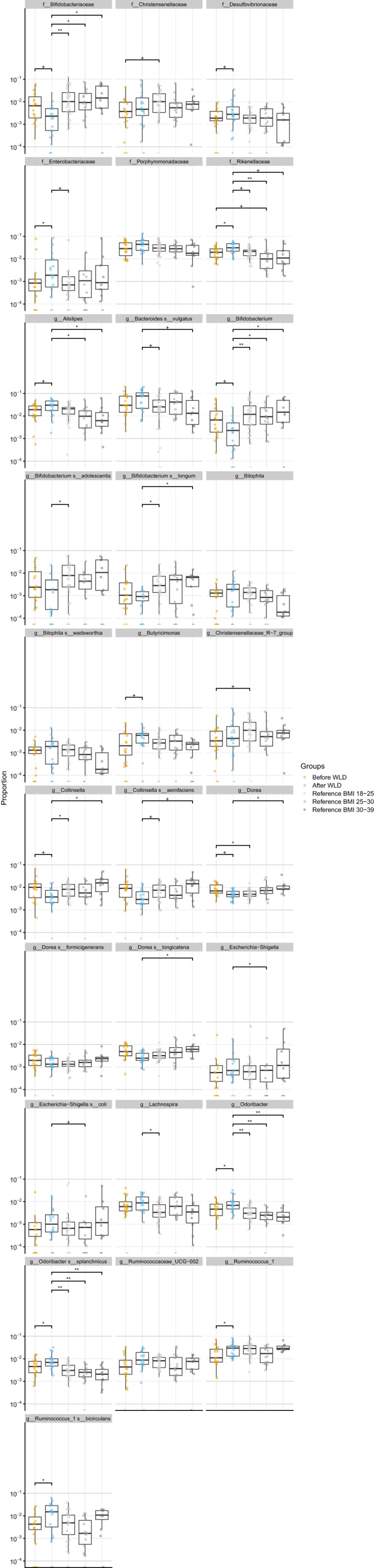
Abundance of significantly altered (uncorrected *p* < 0.05) taxa after WLD in comparison with reference groups

Starting first from higher taxonomic levels, the relative abundance of members of the families Enterobacteriaceae, Rikenellaceae, and Desulfovibrionaceae increased significantly after WLD (Figure 5, Figure A4), while the abundance of Bifidobacteriaceae was reduced.

From the dominant genera, the abundance of *Prevotella* decreased, while the abundance of *Bacteroides* increased, yet both changes were statistically insignificant (*p* ≥ 0.1 for both, data not shown). A more pronounced response was associated with an increase in *Odoribacter* (only species is *O*. *splanchnicus*) and *Ruminococcus*_1 (only species *R. bicirculans*). Additionally, the relative abundance of *Alistipes* and *Butyricimonas* increased, while the abundance of *Bifidobacterium*, *Collinsella*, and *Dorea* decreased.

Among the Enterobacteriaceae, WLD supported the growth of *Escherichia*, mainly *E*. *coli* (uncorrected *p* < 0.05). Another bacteria that was supported during WLD was (Figure 5, Figure A4) *Bilophila wadsworthia*. No other taxonomic groups within the *Desulfovibrionaceae* family showed significant differences.

Additionally, the abundance of Christensenellaceae, Porphyromonadaceae, *Lachnospira*, and *Bacteroides vulgatus* increased, but not significantly, after correcting for multiple comparisons (*p* < 0.05, uncorrected *p* > 0.1). At the species level, changes were less pronounced but still noteworthy, for example, the abundance of *B*. *adolescentis*, *B*. *longum*, *C*. *aerofaciens*, *D*. *formicigenerans*, and *D*. *longicatena* was reduced after WLD but not significantly (*p* < 0.05, uncorrected *p* > 0.1).

**FIGURE A5:**
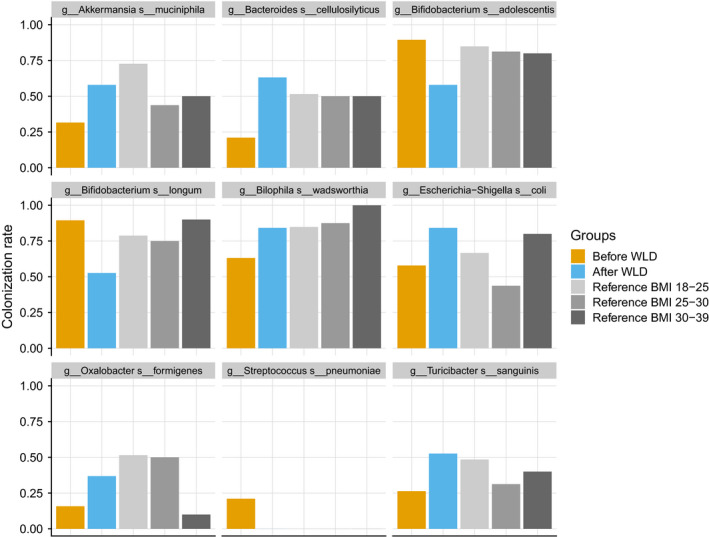
Prevalence of species (|delta prevalence| >3 between before and after WLD) in the test and reference groups

Analysis of presence/absence data at the species level showed that several bacteria were less or more frequent after WLD. The most striking example was observed in the case of cellulose‐degrading *B*. *cellulosilyticus*, which was more prevalent after the intervention and detected (>0.01%) in 12/19 samples after WLD compared to 4/19 samples before the intervention (Figure A5). Additionally, the colonization rate of *A*. *muciniphila*, *B*. *wadsworthia*, *E*. *coli*, *O*. *formigenes*, and *T*. *sanguinis* increased, while the prevalence of *B*. *adolescentis* and *B*. *longum* decreased after WLD.

**FIGURE A6:**
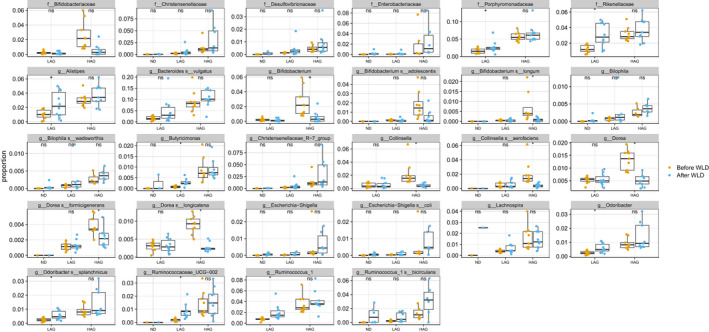
Abundance of significantly altered (uncorrected *p* < 0.05) taxa before (beg) and after (end) WL diet

**FIGURE A7:**
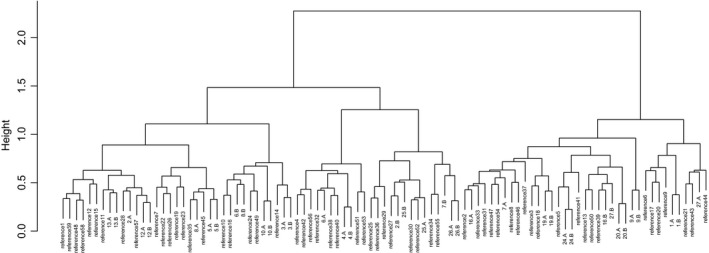
Ward's agglomerative hierarchical clustering on a Bray–Curtis distance matrix

Participants in the WLD group were divided into three subgroups based on their initial abundance of significantly altered taxa: not detected, low, and high abundance groups (ND, HAG, and LAG, respectively). This analysis revealed that changes in bacterial levels depend on starting levels which means that bacteria in the LAG group increased more than in the HAG group. For example, abundances of Porphyromonadaceae, Rikenellaceae (*Alistipes*), *Butyricimonas*, Ruminococcaceae_UCG−002, *Ruminococcus_1*, and *Odoribacter splanchnicus* were significantly increased after WLD in the LAG group compared to insignificant changes in the HAG group (Figure A6). In the case of bacteria, which decreased after WLD, reduction of *Bifidobacterium*, *Collinsella aerofaciens*, and both *Dorea* species was significant in the HAG group, while significant changes were not observed in the LAG group (Figure A6).

### 3.3 Comparison of pre‐ and post‐intervention time points to normal weight, overweight, and obese reference groups

To investigate how WLD altered bacteria compared to the wider population, pre‐intervention and post‐intervention time points were compared with normal weight, overweight, and obese reference groups. Further analysis included only taxa, which were significantly altered in the intervention. No significant difference in bacterial abundances was detected between the obese reference group and the WLD group (Figure 5c, Figure A4). On the other hand, compared to the normal weight reference group, the abundance of *Christensenellaceae* and *Dorea* were lower and higher, respectively, in the WLD group. Overall, most of the taxa, which were significantly shifted in response to WLD, also exhibited distinct relative abundances in post‐WLD samples compared to bacterial levels in reference groups. Most coherent differences between post‐WLD samples and reference groups were detected among *Rikenellaceae* and *O*. *splanchnicus*, which were significantly increased compared to their populations in all reference groups. Similarly, a decline in the abundance of Bifidobacterium was observed in post‐WLD samples compared to that in all reference groups. In addition, post‐interventional levels of *B*. *vulgatus* and *Enterobacteriaceae* were higher than those in the normal weight reference group. The abundance of *B*. *vulgatus* was also higher in the post‐WLD group compared to that in the overweight reference group. In contrast, the abundance of *C*. *aerofaciens* was significantly lower after WLD compared to that in the normal weight and overweight reference group. Although abundances of Porphyromonadaceae, *Bilophila*, *Butyricimonas*, Ruminococcaceae_UCG‐002, *B*. *wadsworthia*, *D*. *formicigenerans*, and *R*. *bicirculans* were elevated after WLD, their abundances remained similar to those observed in all other reference groups.

## 4 DISCUSSION

This study evaluated the effect of a low‐carbohydrate high‐fat weight loss diet (WLD) on the intestinal microbiota of overweight/obese subjects. We specifically analyzed the macronutrient, fatty acid, and dietary fiber composition of appointed WLD to elaborate the effect of weight loss on fecal microbiota and gastrointestinal (GI) symptoms. Another goal of this study was to compare microbiota between WLD and reference groups (normal weight, overweight, and obese individuals).

Consumption of dietary fiber (DF) in a habitual diet has been well characterized by food sources (O’Neil et al., 2012), but weight reduction diets are poorly defined and only provide information about total DF, non‐starch polysaccharides, or resistant starch (RS) content (David et al., 2014; Fava et al., 2013; Santacruz et al., 2009; Walker et al., 2011). To date, the most detailed dietary analysis of fiber intake in a habitual diet was conducted by Munch Roager et al. (2019), who analyzed the effects of wholew grain and refined grain diets on adult human fecal microbiota. They measured the RS, arabinoxylan, and monosaccharide composition of whole‐grain and refined grain products in the diets utilized in the study. In another study, the same group investigated the effects of a low‐gluten diet on fecal microbiota and analyzed the carbohydrate composition of DF in representative meals of the diets utilized in the study (Hansen et al., 2018). They observed that arabinoxylan‐rich cereals were important to keep sufficient levels of bifidobacteria in the fecal microbiota (see below).

In our study, a diet analysis revealed that DF content was moderate and slightly below the recommended value of 12.6 g/1000 kcal (Øverby et al., 2013) and is comparable with the DF content in the reference group with a non‐statistically significant upward trend. Because plants contain a wide variety of DFs, we formed 11 main categories and 45 subcategories of foods to characterize the specific fiber composition of foods. From the main categories, the most abundant fiber source was vegetables, which provided approximately 50% of the total DF intake and subsequently determined the cellulose‐rich nature of WLD. The second richest source of DF was fruits, which significantly increased the amount of pectin and lignin content in WLD. Because cereal consumption was low in WLD, it resulted in a low‐to‐moderate amount of arabinoxylan and β‐glucan in the diet.

In our study, before introducing the WLD regime, the GI symptoms of subjects varied from low Bristol Stool Scale Score (BSS) to high BSS, and many reported frequent flatulence. After the four‐week intervention, these conditions normalized thereby demonstrating a positive effect of this WLD on GI symptoms. These effects can be explained by the high amount of vegetable fibers, for example, cellulose, pectin, and lignin in the WLD plan.

Previous weight‐loss interventions have shown subject‐specific deviations in community composition and considerable alterations in specific bacterial abundances (Ott et al., 2017). In our study, using NMDS analysis, we show that the microbial communities within participants drifted during the intervention and in some cases displayed large changes indicating more significant alterations in the microbial communities. According to the hierarchical clustering, our WLD intervention did not hamper subject‐wise clustering, which has been also shown by Salonen et al. (2014).

Although baseline fecal microbiota had similar abundance profiles compared with reference groups, we observed a lower abundance of *Christensenellaceae* and a higher abundance of *Dorea* compared to the normal weight reference group. These bacteria are known to correlate with BMI (Goodrich et al., 2014). After four weeks of WLD intervention, the enterotype status and α‐diversity were mostly unchanged. However, specific changes in the microbiota were observed, for example, a decrease in the number of *Collinsella*, *Coprococcus*, and *Dorea* species. These results correspond with results from other weight loss studies and dietary interventions, where the α‐diversity (Ott et al., 2017) or enterotype status (Wu et al., 2011) were not affected by short‐term calorie reduction yet an increase in α‐diversity has been reported after long‐term weight loss (Liu et al., 2017).

Our study corroborates previous findings concerning the reduction of bifidobacteria on carbohydrate‐limited hypocaloric diets (Duncan et al., 2007; Salonen et al., 2014; Santacruz et al., 2009). However, an increased abundance of *Bifidobacterium* has been observed on a moderate carbohydrate and fiber‐rich weight reduction diet (Ott et al., 2017). This could be explained by contrasting the macronutrient profiles of the applied diets because the WLD intervention in our study was limited in fiber and carbohydrate content compared to the dietary regime applied by Ott et al. (2017). In studies where diets are aimed to maintain body weight, supporting effect to increase the abundance of bifidobacteria by high‐carbohydrate diets has been shown in comparison with high‐fat diets (Fava et al., 2013). However, a decline in bifidobacteria has been observed also in weight loss intervention on a macronutritionally balanced diet (Santacruz et al., 2009), gluten‐free diet (Palma et al., 2009), and low‐gluten intervention diet (Hansen et al., 2018). Thus, the reduction of *Bifidobacterium* levels after WLD intervention can be attributed to the low intake of cereal grains and starchy vegetables. There is some evidence to support that bifidobacteria are supported by arabinoxylan oligosaccharides, although only a few studies show that long‐chain arabinoxylan is bifidogenic (Hopkins et al., 2003; Monteagudo‐Mera et al., 2018; Truchado et al., 2017). The growth of bifidobacteria is supported by dietary fructans (Dewulf et al., 2013), which are enriched in the wheat endosperm. In WLD, the intake of arabinoxylan and fructan was low‐to‐moderate but comparable to reference group values, and the nature of food components suggests that RS intake could have been low, thus potentially limiting the growth of bifidobacteria. Studies that compare the microbiota of normal weight and obese subjects have shown contrasting results regarding *Bifidobacterium*, which has been associated with both high (Selma et al., 2016; Sepp et al., 2013; Verdam et al., 2013) and low BMI (Ignacio et al., 2016; Korpela et al., 2017; Santacruz et al., 2010) in adults and children. Our study supports the idea that the abundance of bifidobacteria is not conditionally dependent on weight or weight loss and is rather related to fiber type and carbohydrate content in WLD.

Another interesting shift after WLD intervention is an increase in *R*. *bicirculans*. This trend has been also observed in a high‐protein low‐fat weight reduction diet (Salonen et al., 2014). It has been suggested that *R*. *bicirculans* selectively utilizes certain hemicelluloses, especially β‐glucans and xyloglucan (XyG) (Wegmann et al., 2014). Vegetables, especially leaves are rich in XyG, which could also explain the increase in the prevalence of *B*. *cellulosilyticus* on vegetable‐rich low‐cereal WLD (McNulty et al., 2013; Williams et al., 2017).

*Collinsella* and *Dorea* species, which were both reduced in WLD, have been associated with metabolic diseases (Candela et al., 2016; Duvallet et al., 2017; Gomez‐Arango et al., 2018; Goodrich et al., 2014; Lahti et al., 2013; Liu et al., 2017; Zupancic et al., 2012). *C*. *aerofaciens* levels have been shown to decrease on a high‐protein low‐fat weight reduction diet (Walker et al., 2011). Diet‐specific effects on *C*. *aerofaciens* have not yet been elucidated: an increase in prevalence has been observed after a high‐cereal‐grain diet (Foerster et al., 2014) and reduced abundance after vegetable and whole‐grain fiber‐rich fruit‐free diet (Candela et al., 2016). Nutritional studies have shown that low‐gluten intervention reduces the abundance of *Dorea* (Hansen et al., 2018), which agrees with our results because consumption of DF from wheat, rye, and barley during WLD was minimal. Biochemical tests have shown that both *Collinsella* and *Dorea* species exhibit low‐carbohydrate fermentation while the latter species can consume sugars derived from arabinoxylan or fructose (Kageyama et al., 1999; Taras et al., 2002).

Enrichment of *Butyricimonas* and *Rikenellaceae* in lean subjects, negative correlation with BMI and triglyceride levels indicates that these taxa may promote health or contribute to the prevention of obesity (Goodrich et al., 2014; McNulty et al., 2013). Our study supports this idea because these taxa increased after WLD intervention. Furthermore, a high abundance of butyric‐acid‐producing *Butyricimonas* has been associated with normal weight and diets high in animal protein and saturated fats (Garcia‐Mantrana et al., 2018).

High‐fat diets have been previously associated with increased bile release (Cummings et al., 1978; David et al., 2014), while weight reduction diets can reduce serum bile acid (BA) (Biemann et al., 2016; Jahansouz et al., 2016; Straniero et al., 2017) and total fecal BA concentration (Kudchodkar et al., 1977). On the other hand, fecal BA concentrations were not altered during dietary weight loss therapy (Damms‐Machado et al., 2015) but were reduced with a low‐fat hypocaloric diet supplemented by high fiber (Reddy et al., 1988). Even though we did not analyze the BA concentrations in feces or plasma, the abundance of several bile‐tolerant bacteria increased during WLD such as *Rikenellaceae* (*Alistipes*), *Odoribacter splanchnicus*, and *Bilophila wadsworthia*, which indicates that bile concentration may have increased in the GI tract. An increase of *B*. *wadsworthia* on a high‐fat diet has also been observed in other studies (David et al., 2014).

## 5 CONCLUSIONS

This study investigated changes in fecal microbiota during significant weight loss on a high‐fat diet. In contrast with most weight loss studies, we characterized the DF sources and estimated the specific DF intake in the diets used which provides an additional layer of data to link microbiota alterations with diet. To our knowledge, this is the first publication that characterizes specific fiber intake and DF intake quantitatively by food subcategories in a weight loss study based on food composition data. WLD intervention both reduced BMI and improved GI symptoms. High vegetable intake increased the levels of cellulose and low‐cereal intake reduced the levels of arabinoxylan and β‐glucan content in the diet, which were accompanied by shifts in microbiota such as a reduction in the abundance of bifidobacteria. WLD supported the growth of bile‐resistant bacteria, while the abundance of bacteria associated with inflammation was reduced. We conclude that the dietary intake of different fibers and the initial abundance of bacteria in the microbiota (low or high abundant groups) should be taken into account when analyzing the impacts of a weight reduction diet.

## CONFLICT OF INTEREST

None declared.

## AUTHOR CONTRIBUTIONS

**Madis Jaagura:** Conceptualization (supporting); Data curation (equal); Methodology (supporting); Software (lead); Validation (equal); Visualization (lead); Writing‐original draft (lead); Writing‐review & editing (equal). **Ene Viiard:** Conceptualization (equal); Writing‐review & editing (supporting). **Kätrin Karu‐Lavits:** Data curation (equal). **Kaarel Adamberg:** Conceptualization (lead); Funding acquisition (lead); Methodology (lead); Supervision (lead); Validation (equal); Writing‐review & editing (equal).

## ETHICS STATEMENT

The study protocol was approved by the Tallinn Medical Research Ethics Committee (TMEK no 1631). Informed consent was obtained from all subjects involved in the study.

## Data Availability

All data are provided in full in this paper and its appendices. The raw sequences obtained were demultiplexed and uploaded to the European Nucleotide Archive (ENA) under the accession number PRJEB35687: https://www.ebi.ac.uk/ena/browser/view/PRJEB35687

## 1

**TABLE A1:** Literature overview of weight loss interventions

Tested diets	Main TDF sources	Main results	Reference
**M**—energy maintenance diet (~3100 kcal, ~28 g NSP/day, 52% C, 12% P, 35% F) 3 days **HPMC**—ad libitum (~1800 kcal/day, ~12 g NSP/day, 35% C, 30% P, 35% F) 4 weeks **HPLC**—ad libitum (~1700 kcal/day, ~6 g NSP/day, 4% C, 30% P, 66% F) 4 weeks.	M/HPMC/HPLC: not available/unknown	HPMC and HPLC (vs M): fecal acetate↓, butyrate↓, total SCFA↓, Roseburia/E.rectale↓, bifidobacteria↓ HPLC (vs HPMC): fecal butyrate↓	Duncan et al., (2007) *N* = 19
**LC**—ad libitum (~1600 kcal/day, ~13 g TDF/day, 5% C, 35% P, 58% F) 8 weeks **HC**—ad libitum (~1500 kcal/day, ~32 g TDF/day, 46% C, 24% P, 28% F) 8 weeks	LC: >2.5 cups green vegetables, 40 g nuts HC: 40 g bran cereal, 35 g whole grain bread, 300 g fruit, >2.5 cups green vegetables, potato, pasta, rice, bean lentils, 20 g nuts	LC (vs HC): fecal output↓, defecation frequency↓, fecal butyrate↓, total fecal SCFA↓, bifidobacteria↓	Brinkworth et al., (2009) *N* = 91
**M**—energy maintenance diet (~2800 kcal/day, ~22 g NSP/day, 51% C, 12% P, 37% F) 4 weeks **HPMC**—(~2000 kcal/day, ~13 g NSP/day, 36% C, 28% P, 37% F) 4 weeks **HPLC**—(~1900 kcal/day, ~9 g NSP/day, 4% C, 30% P, 66% F) 4 weeks.	M/HPMC/HPLC: varied	HPMC (vs M): fecal isovalerate↑, isobutyrate↑ HPLC (vs M): fecal butyrate↓, total SCFA↓, isovalerate↑, isobutyrate↑, *Roseburia*/*E*.*rectale*↓, *Bacteroides*↓	Russel et al. (2011) *N* = 17
**M**—maintenance diet (~3300 kcal/day, ~33 g TDF/day, 52% C, 13% P, 35% F) 1 week **WL**—weight loss diet (~2000 kcal/day, ~28 g TDF/day, 41% C, 30% P, 29% F) 3 weeks	M: not available/unknown (RS 5 g/day, soluble NSP 4 g/day, insoluble NSP 23 g/day) WL: not available/unknown (RS 3 g/day, soluble NSP 3 g/day, insoluble NSP 22 g/day)	WL (vs M): *C*. *aerofaciens*↓, *Roseburia*↓, *Oscillibacter*↑ (Walker) WL (vs M): fecal acetate↓, propionate↓, butyrate↓, succinate↓, *Bifidobacterium*↓, *P*. *cinnamivorans*↓, *B*. *vulgatus*↑ (Salonen)	Walker et al., (2011)^a^ Salonen et al., (2014)^a^ *N* = 14
**Before intervention** (~2100–2400 kcal/day, ~17–18 g TDF/day, 42–43% C, 19% P, 38–40% F) 10 weeks **WL**—weight loss diet (~1400–1500 kcal/day, ~18–22 g TDF/day, 45–47% C, 22–23% P, 32–34% F) 10 weeks	WL: Vegetables, cereals, fruits, and legumes	*B*. *fragilis*↑, *Lactobacillus*↑, *C*. *coccoides*↓, *B*. *longum*↓, *B*. *adolescentis*↓	Santacruz et al., (2009) *N* = 36
**Before intervention** (~1700 kcal/day, ~15 g TDF/day, 45% C, 17% P, 38% F) **VLCD**—(~800–900 kcal/day, ~15 g TDF/day, 51% C, 26% P, 23% F) 4 weeks	Not available/unknown, supplementary vegetables	HDL↓, LDL↓, TG↓, CRP↓, LBP↓, gut paracellular permeability↓, *A*. *rectalis*↓, *R*. *faecis*↑, *Bifidobacterium* sp.↑, *Blautia* sp.↑, *A*. *hadrus*↑	Ott et al., (2017) *N* = 20

TDF (total dietary fiber) includes both non‐starch polysaccharides (NSP) and resistant starch (RS).

Abbreviations: %E, Percentage from energy; C, carbohydrates; F, fat; P, protein.

^a^ Other tested diets not described

**TABLE A2:** GI disturbances frequency and intensity

	Before WLD							After WLD				
	Co	D	B	F	Cr	S‐P	Co	D	B	F	Cr	S‐P
Frequency of gastrointestinal symptoms												
Daily	1	1	2	7	0	0	0	0	1	1	0	0
Weekly	3	3	4	6	0	0	3	2	3	8	0	1
Monthly	6	5	4	1	2	5	6	5	3	3	1	3
<10 × year	4	5	3	0	2	4	4	6	5	1	3	4
Never	0	0	1	0	10	5	1	1	2	1	10	6
Intensity of gastrointestinal symptoms												
Strong^a^	1	0	1	0	0	0	0	0	0	0	0	0
Moderate	3	3	3	8	1	1	2	2	2	6	2	2
Mild^b^	8	9	7	6	2	6	10	9	8	7	2	4
No symptoms	2	2	3	0	11	7	2	3	4	1	10	8

Abbreviations: B, bloating; Co, constipation; Cr, cramps; D, diarrhea; F, flatulence; S‐P, stomach pain.

^a^ Disturbs considerably.

^b^ Does not disturb.
